# Overexpression of *PLIN1* Promotes Lipid Metabolism in Bovine Adipocytes

**DOI:** 10.3390/ani10111944

**Published:** 2020-10-22

**Authors:** Shijun Li, Sayed Haidar Abbas Raza, Chunping Zhao, Gong Cheng, Linsen Zan

**Affiliations:** 1 College of Animal Science and Technology, Northwest A&F University, Yangling 712100, China; lishijun1990cn@163.com (S.L.); haiderraza110@nwafu.edu.cn (S.H.A.R.); zhao.chunping@foxmail.com (C.Z.); chenggong@nwafu.edu.cn (G.C.); 2 National Beef Cattle Improvement Center, Northwest A&F University, Yangling 712100, China

**Keywords:** cattle, *PLIN1* gene, bovine adipocytes, adenovirus, RNA-seq, differentially expressed genes

## Abstract

**Simple Summary:**

In bovine preadipocytes, the overexpression of the *PLIN1* gene promotes the expression of *FASN*, *PPARγ*, *ACC*, *LPL*, *FABP4*, *DGAT2*, and *C/EBPβ* at the mRNA level, inhibiting the expression of fat metabolism genes such as *PLIN2* and *ATGL*. Furthermore, after overexpression of *PLIN1*, the oil red O-staining results showed that the number of lipid droplets (LDs) in fat cells, their volume and triglyceride content were increased. The elevation in triglyceride content indicates that *PLIN1* can promote the accumulation of triglyceride in bovine preadipocytes and has an important regulatory role in fat metabolism. After transfection with the adenovirues Ad-PLIN1 and Ad-NC (empty virus), RNA-seq was used to analyze the differentially expressed genes in bovine preadipocytes. A total of 1923 differentially expressed genes were detected, and GO and KEGG signaling pathways were analyzed for differentially expressed genes.

**Abstract:**

Perilipin 1 (PLIN1) is a protein encoded by the *PLIN1* gene in eukaryotes. PLIN1 is a member of the PAT protein family, a family of proteins related to lipid droplet (LD) surface proteins. PLIN1 phosphorylation plays a vital role during fat metabolism of adipose tissue lipolysis and fat storage in adipocytes. However, to further explore the regulation of the *PLIN1* gene on the proliferation, differentiation and lipid metabolism of bovine adipocytes. In this study, the mRNA expression of *PLIN1,* at day six, was the highest during bovine adipocyte differentiation. Moreover, *PLIN1* can promote the proliferation and differentiation of preadipocytes in cattle. On the sixth day, after transfection with, and overexpression of, the *PLIN1* gene in bovine preadipocytes via adenovirus, cell samples were collected, and transcriptome sequencing was performed. A total of 1923 differentially expressed genes were detected. Through GO and KEGG pathway analysis, the differentially expressed genes were established to be mainly enriched in the AMPK, Wnt, and PPAR signaling pathways related to fat proliferation and differentiation. In conclusion, at the transcriptional level, *PLIN1* plays an important role in regulating fat proliferation and metabolism. Additionally, the sequencing results screened new differentially expressed genes related to fat metabolism, providing theoretical support for molecular breeding of Qinchuan beef cattle.

## 1. Introduction

For beef cattle, the marbling grade of meat is an important criterion reflecting the quality of beef [1], and marbling is the major determinant of consumer acceptability [2]. Qinchuan cattle is an indigenous Chinese breed and one of the five finest yellow cattle in China. It is mainly produced in the Guanzhong Plain of Shaanxi. This variety has the advantage of fast growth rate, but the meat quality traits, such as intramuscular fat content are still not comparable with the exotic varieties like Wagyu. The marble pattern of beef is mainly affected by intramuscular fat content [3]. In mammals, fat is predominantly stored as subcutaneous, visceral, or intramuscular fat. Intramuscular fat content is an important factor in determining meat quality [4,5]. Fat deposition in muscles involves a series of processes, such as preadipocyte proliferation, preadipocyte differentiation, and lipid metabolism, and is regulated by related functional genes [6]. Therefore, it is particularly important to study the mechanism of fat formation and the regulation of genes related to meat quality traits at the molecular level. Lipid droplets (LDs) are common dynamic cellular organelles that contain unique surface proteins in most mammalian cells and are present in various cell types. The PAT family of proteins not only play pivotal roles in the formation and degradation of lipid droplets, but also regulates TG (triacylglycerol) packaging. The PAT family is comprised of five different lipid droplet proteins in mammals: Perilipin, adipose differentiation-related protein (ADRP), tail-interacting protein of 47 kilo Daltons (TIP47), S3-12, and OXPAT [7]. The PAT family proteins play important roles in regulating intracellular lipid storage, and are widely present in many species [8]. All PAT proteins exhibit sequence similarity and possess the ability to bind intracellular LDs. The differences between various PAT proteins affect their functions in cells. However, all PAT proteins may perform the function of regulating LDs within the cellular environment [9].

The adenovirus AdEasy system is a simple and convenient adenovirus recombination system composed of an adenovirus shuttle vector (pAdTrack or pShuttle) and a backbone vector (pAdEasy), which was constructed by He in 1998 [10]. Firstly, a recombinant adenovirus plasmid can be produced by inserting a target gene into a shuttle vector (e.g., pAdTrack-CMV) and then inserting a recombinant adenovirus. After transfection of these plasmids into a mammalian packaging cell line, virus production is conveniently carried out through green fluorescent protein.The overexpression of, and interference with, target genes to detect their effects at the cellular, mRNA, and protein levels are important methods for studying gene function. Adenovirus vectors are currently the most effective transgenic vectors, and they have the advantages of high infection efficiency, low pathogenicity, and high titer [11]. Adenovirus vectors can be used in cells of different periods and are widely employed in human gene therapy [12]. The effect of the *PLIN1* gene on lipid metabolism in bovine preadipocytes is rarely reported. PLIN1 is one of the most abundant protein on LDs in the adipocytes. The perilipin 1 protein coats the outer layer of LDs to prevent lipases in the body, such as hormone-sensitive lipase (HSL) and lipase activator (ATGL), from entering LDs [13]. In the adipose tissue of *PLIN1*-null mice, some sterol biosynthetic enzymes were downregulated [14], indicateing that *PLIN1* deficiency may affect the sterol biosynthetic pathway in steroidogenic cells as *PLIN1* activates HSL. Lipolysis in the adipose tissue was significantly reduced, and the adipose tissue was enhanced in *PLIN1*-null mice [15]. Studies have compared different levels of lipolysis in *PLIN1*-null and wild-type mice and found that *PLIN1*-null adipocytes had reduced catecholamine-stimulated lipolysis and increased rates of constitutive (unstimulated) lipolysis [16]. *PLIN1* is essential during the liposecretion process, and it can be expressed on the LD surfaces in white and brown mature adipocytes [17]. Studies have shown that PLIN1 is an important regulator of lipolysis and protects LDs from basal lipolysis. However, when fat cells are stimulated to initiate lipolysis, PLIN1 regulates lipohydrolase in LDs to complete the hydrolysis [18]. Accordingly, *PLIN1* knockout mice are lean and protected from diet-induced obesity [19]. Importantly, the absence of *PLIN1* causes dysregulation of adipogenic signaling, impairing the development and differentiation of adipose cells [20]. In *PLIN1*-/- mice, both adipogenic transcription factors, such as PPARγ and C/EBPs, and lipolysis-related enzymes, such as HSL and ACC1, were downregulated [21]. Previous studies have revealed that *PLIN1* plays a key role in adipogenesis and LD formation, and that *PLIN1* may participate in the process of the liposecretion process of mature adipocytes [16,22]. Proliferation and liposecretion are regulated through key pathways, including Akt, Erk, and Wnt pathways [21]. In particular, TGFβ is mediated through various PLIN1 proteins which have specific activities either as differentiation or proliferation modulators [23]. However, the molecular mechanisms involved in the regulation of lipid metabolism by *PLIN1* remain unclear in bovine adipocytes. The *PLIN1* gene in Qinchuan cattle may feature prominently in fat proliferation and differentiation. Therefore, this study investigated the differentially expressed genes (DEGs) and related signaling pathways through transcriptome sequencing of cell samples. In addition, we studied the functional network of LD formation and fat metabolism, and speculated on the potential pathways that regulate fat metabolism. These findings have improved our understanding of lipid metabolism and provide theoretical support for molecular breeding of Qinchuan beef cattle in Qinchuan.

## 2. Materials and Methods

### 2.1. Sample Information

The animal samples for the experiments were approved in accordance with code NWAFUC/110 of Northwest A&F University, China, and the use of experimental animals followed all guidelines of the organization and government.

### 2.2. Construction of Interference and Overexpression Adenovirus Vectors of PLIN1

An overexpression adenovirus vector, with a full-length bovine *PLIN1* gene and coding sequence, was developed as previously described and termed as Ad-PLIN1 [23]. Firstly, the full-length coding sequence of the *PLIN1* gene was amplified and then, without the site mutation sequence, the pAd-Track shuttle vector was selected and cloned. The sequence of *PLIN1* was cloned into the pAd-Track shuttle vector. The shutter vector was linearized by *Pme I* restriction enzyme (NEB, USA) and recombined with the pAdEasy-1 vector in BJ5183 competent cells to achieve the homologous recombination of shuttle vector and skeleton vector in cells, and the adenovirus recombinant pAd-PLIN1 was obtained. Finally, the pAd-PLIN1 expression vector was removed by the extraction of gel after it was digested with the Pac I restriction enzyme (NEB, USA) and converted into HEK 293A cells using the liposome method to be packed into an adenovirus (Ad-PLIN1). The National Beef Cattle Development Center has retained and developed negative control of adenovirus (Ad-NC).

To construct an interference adenovirus vector for negative control oligonucleotides (sh-NC) and unique short-hairpin RNA (shRNA) for bovine PLIN1 mRNA, this study used online application called BLOCK-iT Designer RNAi (Thermo fisher scientific, Waltham, MA, USA). Subsequently, we cloned shRNAs into the pENTR/U6 RNAi input vector and the psiCHECK-II reporter device (Promega, Madison, WI, USA) to detect interference efficiencies of the pENTR/U6 RNAi input vector. shRNA-1337 (Table 1) was chosen with a maximum intervention quality of 76.2 percent, which is acceptable for adenovirus packaging. The pENTRTM/U6-shRNA-1337 was then recombined with the pAd/PL-DEST™ disruption vector. Pursuant to LR Clonase Control II Enzyme (Invitrogen, Carlsbad, CA, USA), transmitted vector (Invitrogen, Carlsbad, CA, USA) in vitro. Adenovirus plasmid recombinant sh-PLIN1 was obtained and transferred to HEK 293A cells to enable adenovirus (sh-PLIN1) to be packaged.

### 2.3. Isolation, Culture, and Differentiation of Bovine Preadipocytes

Bovine preadipocytes were collected from five-day-old healthy Qinchuan calves according to the method described in the previous literature [24,25]. First, the fat tissue near the *longissimus dorsi* muscle was taken from healthy calves under aseptic conditions. The fat tissue was first washed with 75% ethanol, cut into small tissue pieces, and transferred into a 15 mL centrifuge tube. The sample was washed with 1× PBS three times and immediately transferred to the cell culture room. The cells were inoculated into T75 culture flasks at a density of 5 × 10^4^ cells/cm^2^, and cultured in an incubator (Thermo Scientific, Waltham, MA, USA) at 37 °C in saturated water with 5% CO_2_. After centrifugation, the preadipocytes were inoculated in an appropriate amount of complete Dulbecco’s modified Eagle medium (DMEM/F12) (Gibco, Shanghai, China). After being cultured until contact inhibition for 2 d, the induction solution was replaced with DMEM/F-12 supplemented with 10% FBS (PAN-Biotech, USA), 1% penicillin/streptomycin, 0.5 mM IBMX (Sigma, St. Louis, MO, USA), 1 μM DEX (Sigma, St. Louis, MO, USA), and 5 μg/mL bovine insulin (Sigma, St. Louis, MO, USA) to induce differentiation. The appearance of lipid droplets can be observed by the inverted fluorescence microscope (Olympus IX71, Tokyo, Japan).

### 2.4. Tissue Collection and Quantitative Reverse-Transcription PCR (RT-PCR) Analysis

For tissue collection, 12 tissue samples were obtained from 3 adult male Chinese indigenous Qinchuan cattle, including- subcutaneous fat, longissimus thoracis, and kidney. Total RNA was extracted from the tissue using TRIzol^TM^ Reagent (Invitrogen, Carlsbad, CA, USA) and then cDNA libraries were constructed using Prime Script^TM^ RT reagent kit (Takara, China). RT-PCR was performed following the protocol of the SYBR Green PCR Master Mix kit (Takara, Beijing, China) and 7500 System (Applied Biosystems, Foster City, CA, USA). The expression levels of target mRNA were calculated using the 2^−ΔΔCt^ method [26].

### 2.5. Western Blotting

Total protein was extracted from bovine adipocytes using a lysis buffer with protease inhibitors. Protein samples were added to a 12% SDS-PAGE gel at 20 μg per well for electrophoretic migration; thereafter, the protein bands on the SDS-PAGE gel were transferred to a polyvinylidene difluoride (PVDF) membrane. The membrane was then incubated with a primary antibody was incubated overnight at 4 °C. The membrane was then washed with phosphate buffer (PBST) for three times and incubated with a secondary antibody for 2 h at room temperature. The membrane was then exposed to detect luminescence signals using a ChemiDoc. XRS+ System (Bio-Rad, Hercules, CA, USA).

### 2.6. Oil Red O Staining and Cellular Triglyceride Determination

Cellular triglyceride levels were determined as previously described [27,28]. Bovine preadipocytes were inoculated into six-well cell culture plates. After 6 d of induction of differentiation, the cells were collected by trypsinization, centrifugation and then lysed. A total of 0.1 mL of the lysate could be added per 1 × 10^6^ cells proportionally. Standard samples were prepared using 4 mM glycerol standards and distilled water. The triglycerides cleaved from the cells were extracted with a tissue triglyceride assay kit (Applygen Technologies Inc., Beijing, China), and the OD value was then measured at 550 nm using a microplate reader. Finally, the concentration of triglyceride was calculated from a standard curve prepared from the OD value of the standard sample. Triglyceride levels were normalized to the protein concentration of each sample. Finally, the concentration of triglycerides was calculated from a standard curve prepared from the OD value of the standard sample. After 6days of differentiation induction, the culture medium of the cells was discarded. Thereafter, the cells were washed three times with phosphate-buffered saline (PBS), fixed with 4% paraformaldehyde for 30 min. Oil red O working solution was added to cover the bottom of the cell culture dish, and the cells were stained for 30 min at room temperature. The cells images were captured using an Olympus 1 × 71 microscope (Olympus IX71, Tokyo, Japan).

### 2.7. The 5-ethynyl-2-deoxyuridine (EdU) Proliferation Assay

EdU assay was performed using the cell light EdU DNA proliferation kit (RiboBio, Suzhou, China). Bovine preadipocytes were seeded in 24-well cell culture plates. When the cell density reached 50–60% density, the cells were transfected with Ad-NC, Ad-*PLIN1*, sh-NC, or sh-*PLIN1*. After 24 h, the cells were incubated with EdU medium for 2 h. The EdU assay was performed according to the manufacturer’s protocol. The cell images were captured using an Olympus microscope (Olympus IX71, Tokyo, Japan).

### 2.8. Cell Cycle Assay through Flow Cytometry

Bovine preadipocytes were seeded in six-well cell culture plates, and the cells were transfected with Ad-NC, Ad-*PLIN1*, sh-NC, or sh-*PLIN1*. The cells were collected, washed with 1XPBS, and resuspended in 1× PBS. The cell cycle was analyzed through flow cytometry (FACS CantoTM II, BD BioSciences, San Jose, CA, USA) by counting 20,000 cells.

### 2.9. RNA Sequencing (RNA-seq)

The bovine preadipocytes were cultured in 10 cm cell culture plates. Cell samples were collected after induction of differentiation at 6 d. RNAs from three biological replicates of each group were isolated using RNeasy kit (Qiagen China (Shanghai) Co Ltd., Shanghai, China). The cell samples were subsequently sent to Novo Genefor transcriptome sequencing.

### 2.10. Identification of DEGs and Functional Enrichment Analysis

The edgeR package (http://www.r-project.org/) was used to identify DEGs within samples. GO term enrichment analysis was performed using the online Visualization and Integrated Discovery (DAVID) software [29,30] for the three GO categories. In addition, KEGG pathway enrichment analysis was carried out using the KEGG Orthology-Based Annotation System (KOBAS) software [31,32]. Significant enrichment was considered for a corrected *p*-value < 0.05.

### 2.11. qRT-PCR Validation of Key Genes

Total RNA from bovine adipocytes cells was extracted using RNAiso (Takara, Beijing, China). A SYBR^®^ Premix Ex Taq^TM^ II Kit (Takara) was used to carry out qRT-PCR with an ABI 7500 Real-Time PCR system (Applied Biosystems, Foster City, CA, USA). GAPDH served as an internal reference to normalize gene expression levels via the 2^−∆∆Ct^ method [33].

### 2.12. Statistical Analyses

The data were expressed as the mean ± standard deviation and analyzed through analysis of variance (ANOVA) using the Statistical Package for the Social Sciences (SPSS), version 17(IBM, Armonk, NY, USA). The results are shown as mean ± standard error of the mean (SEM).

## 3. Results

### 3.1. Expression of PLIN1 in Bovine Preadipocytes

**Figure 1:**
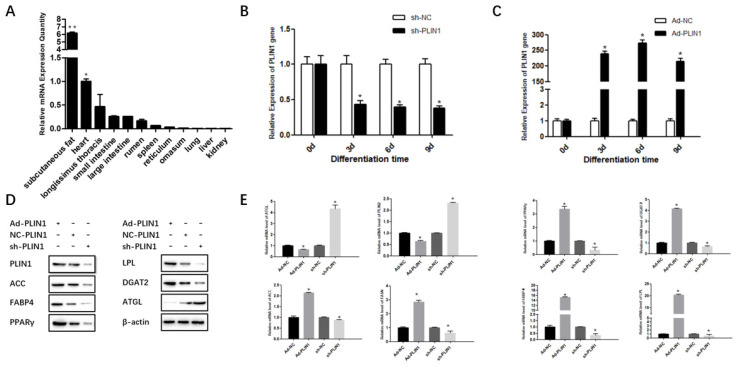
Expression patterns of the *PLIN1* gene during bovine preadipocyte differentiation. (**A**) The relative expression of the *PLIN1* gene was detected by qPCR in12 different bovine tissues. (**B**) After infecting adipocytes with sh-*PLIN1* and sh-NC, the expression of PLIN1 gene decreased in bovine preadipocytes. (**C**) After allowingoverexpression of Ad-*PLIN1* and Ad-NC in adipocytes, the expression of PLIN1 decreased in bovine preadipocytes. (**D**) Effects of overexpression and interference of the *PLIN1* gene on the protein expression levels of lipid metabolism-related genes. (**E**) Effects of overexpression of and interference with *PLIN1* on mRNA expression level of fat-related genes. The values represent mean ± SEM (n = 3). * *p* < 0.05.

To elucidate the function of the *PLIN1* gene in different tissues of Qinchuan cattle, the expression levels of *PLIN1* were determined using qRT-PCR. The expression of *PLIN1* was highest in subcutaneous fat and lowest in the kidney (Figure 1A; *p* < 0.05). In order to systematically research the temporal expression of *PLIN1* during bovine preadipocyte differentiation, this study built the differentiation pattern of small adipocytes, culture the cells within 9 d, and detect the expression level of *PLIN1* every 3 d. The adenoviruses Ad-NC, Ad-*PLIN1*, sh-NC, and sh-*PLIN1* were transfected into bovine preadipocytes. The total RNA of cells at 0 d, 3 d, 6 d, and 9 d was collected. After interfering with the *PLIN1* gene, the mRNA expression of *PLIN1* was reducing (Figure 1B; *p* < 0.05). Moreover, after overexpression of *PLIN1*, the mRNA expression of *PLIN1* increased with an increase in induction differentiation time, and the expression of 6d was the highest (Figure 1C; *p* < 0.05).

Furthermore, the expression levels of *PLIN1*, adipose differentiation marker genes, and lipid metabolism marker genes were detected on the sixth day. The protein expression levels of *PLIN1*, *ACC*, *PPARγ*, *DGAT2*, *FABP4*, *ATGL*, and *LPL* were detected (Figure 1D; *p* < 0.05). The protein expression levels of *PLIN1* were significantly up-regulated when treated with Ad-*PLIN1* and decreased significantly when sh-*PLIN1* was used (Figure 1D; *p* < 0.05). To explore the effect of *PLIN1* on lipid metabolism, the expression levels of genes related to lipid metabolism in adipocytes were determined. After overexpression of *PLIN1*, the mRNA expression levels of *ACC*, *FASN*, *PPARγ*, *DGAT2*, *FABP4*, and *LPL* were significantly increased, and the protein levels of FAS and PPARγ also increased. On the contrary, after interfering with the *PLIN1* gene, *ACC*, *FASN*, *PPARγ*, *DGAT2*, *FABP4*, and *LPL* mRNA expression decreased, and the ACC and FAS protein levels also decreased (Figure 1D,E; *p* < 0.05).

### 3.2. PLIN1 Affected Triglyceride Level and LD Sizes in Bovine Preadipocytes

**Figure 2:**
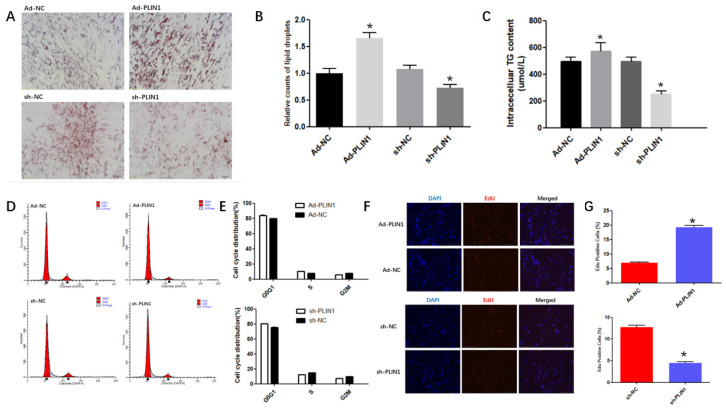
The *PLIN1* gene affected triglyceride levels and LD formation in bovine adipocytes. (**A**,**B**) *PLIN1* overexpression and interference observed by staining bovine adipocytes with oil red. The lipid droplets were counted using the ImageJ software. (**C**) Effects of overexpression and interference of the *PLIN1* gene on triglyceride storage in bovine adipocytes (**D**,**E**) Flow cytometric analysis of *PLIN1* gene in bovine adipocytes. (**F**,**G**) The cells are stained with EdU after four different adenovirus treatments. The cells were counted using the ImageJ software. The values represent mean ± SEM (n = 3). * *p* < 0.05.

This study examinedLD and triglyceride content in adipocytes after overexpression of and interference with *PLIN1*. The number and size of LDs of fat cells were detected via oil red O staining. After overexpresion of *PLIN1*, the number of fat droplets in the fat cells increases and the volume becomes larger, promoting the accumulation of fat droplets in adipocytes. The number of lipid droplets decreased in bovine adipocytes after interference with *PLIN1* (Figure 2A). The LDs were counted using ImageJ software. The relative counts of lipid droplets showed the same result in the staining pictures(Figure 2B; *p* < 0.05). Upon overexpression of *PLIN1*, the triglyceide content in adipocytes was significantly higher than that in the control group, and the content of triglyceride in adipocytes after interference with *PLIN1* was less than that in the control group(Figure 2C; *p* < 0.05).The results show that the *PLIN1* gene plays crucial role in lipid metabolism.

Furthermore, according to the results, interference with *PLIN1* gene expression reduced the percentage of S-phase cells, while up-regulation of *PLIN1* increased the percentage of S-phase cells (Figure 2D–F; *p* < 0.05). To verify the effect of *PLIN1* gene on the proliferation of bovine preadipocytes, EdU staining was used to investigate. Overexpression of *PLIN1* increased the number of EdU-labeled cells. However, downregulation of *PLIN1* significantly decreased the number of EdU-labelled cells (Figure 2G; *p* < 0.05). In general, after interfering with *PLIN1* gene, the mRNA expression of *PLIN1* was reducing.

### 3.3. Study of DEGs in Bovine Adipocytes

**Figure 3:**
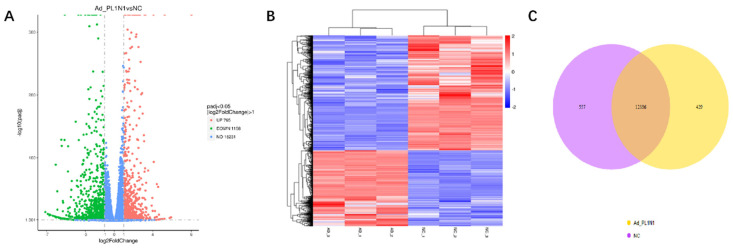
RNA-seq based DEGs between the groups. (**A**) The DEGs represented by volcanic map. The significant DEGs represented as upregulated (red dots) and downregulated (green dots), whereas, blue dots shows the non-significant differential expressed gens. (**B**) The DEGs presented by clustering map. The genes with high expression showed as Red color and genes with low expression were indicated by showing the blue genes. (**C**) The DEGs represented by Venn diagram. Venn diagram showed the overlapping portion by sharing the expression of pattern of the DEGs.

In order to further study the mechanism of the influence of *PLIN1* gene on the differentiation of bovine adipocytes, transcriptome sequencing experiments were carried out. After adding the adenovirus to the bovine adipocytes and culturing them in the differentiation medium for 6 days, cell samples were collected. The studied group was treated with Ad-PLIN1, whereas the control group was treated with Ad-NC. The limma or DESeq2 package was utilized to analyze the raw RNA-seq, and the obtained findings were then validated by RRA (Robust rank aggregation) with adjusted *p*-value < 0.05) using the log_2_FC standard. Based on the RNA-seq results of the Ad-*PLIN1* and Ad-NC treatment groups, a total of 1923 DEGs were detected, in which 765 genes were significantly upregulated and 1158 genes were significantly downregulated (Figure 3A; *p* < 0.05). The genes with high expression showed as red color and genes with low expression were indicated by showing the blue genes (Figure 3B; *p* < 0.05).The Ad-PLIN1 group and the control group (Ad-NC) jointly expressed 12336 genes, the overexpression group specifically expressed 557 genes, and the control group specifically expressed 429 genes (Figure 3C; *p* < 0.05).

### 3.4. GO Terms and KEGG Pathway Analysis of the DEGs

**Figure 4:**
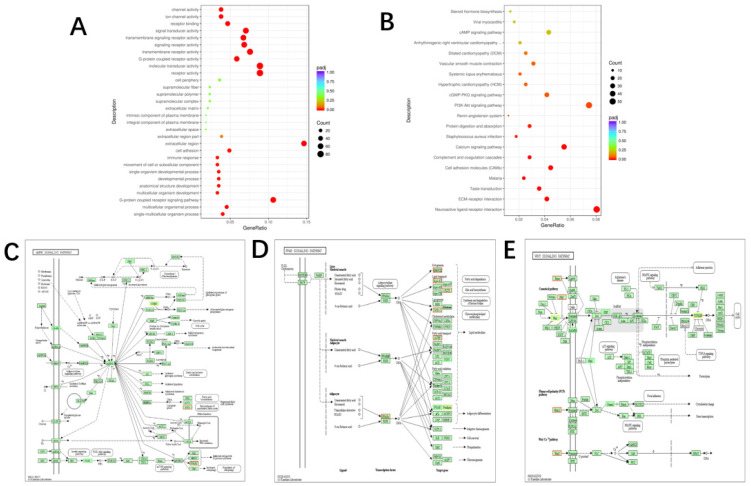
GO terms and KEGG pathway analysis of the DEGs Differentially Expressed Genes. (**A**) The differential expression of differential genes in the three GO terms of biological processes, cell components and molecular functions was sorted and analyzed. The 30 most significant GO terms are enriched. (**B**) The KEGG database was used to analyze the significance of pathway enrichment of differentially expressed genes, and the 20 pathways were significantly enriched. (**C–E**) Differentially expressed genes are enriched in signaling pathways such as AMPK signaling pathway, PPAR signaling pathway, and Wnt signaling pathway, which are related to fat metabolism.

In order to discover the relationship between the DEGs and phenotypic characteristics in adipocyte differentiation, the GO enrichment of DEGs was analyzed (Figure 4A). Among these genes, most were found to be involved in intracellular organelle homeostasis as well as cellular metabolic processes, indicating that different genes are coordinatly involved in biological processes. Furthermore, we classified the DEGs to reveal the enrichment of significant DEGs by using the KEGG database pathways. Based on the KEGG enrichment of DEGs, the majority of genes were found to play a crucial role in metabolic and cancer pathways (Figure 4B). Nonetheless, several DEGs were still associated with fatty acid biosynthesis, such as those of the PPAR, AMPK, and Wnt pathways (Figure 4C–E; *p* < 0.05). There were six differentially expressed genes in the AMPK signaling pathway in different treatment cells, two upregulated genes, *PPARγ* and *SCD1*, and four down-regulated genes, *Ob Rb*, *FBP*, *P13K*, and *AMPK* (Figure 4C; *p* < 0.05). There were eight differentially expressed genes in the PPAR signaling pathway in different treatment cells, and 6 up-regulated genes, namely *HMGCS 2*, *ApoAI*, *PLTP*, *ACBP*, *PPARγ* and *SCD1*; two downregulated genes, *OLR1* and *PGAR* (Figure 4D; *p* < 0.05). There were seven genes differentially expressed in the Wnt signaling pathway in different treatment cells, and four genes are up-regulated, namely *RSPO*, *FRP*, *WNT11*, and *WNT 5*; there were three down-regulated genes, namely *GBP*, *NKD* and *DAAM1* (Figure 4E; *p* < 0.05).

### 3.5. Identification of the DEGs during Promote of Differentiation in Bovine Adipocytes

**Figure 5:**
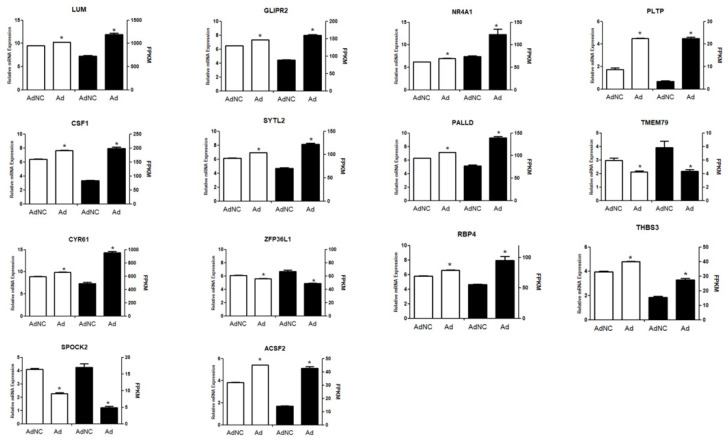
qPCR verification transcriptome sequencing of DEGs. Fourteen DEGs were selected, namely *ACSF2*, *RBP4*, *THBS3*, *LUM*, *GLIPR2*, *CSF1*, *SYTL2*, *NR4A1*, *PLTP*, *PALLD*, *TMEM79*, *CYR61*, *ZFP36L1* and *SPOCK2*, with *GAPDH* as the internal reference gene (Table 2).

To validate the results of RNA-sequencing, fourteen DEGs were selected from the transcriptome sequencing results, and their expression in bovine adipocytes treated with different adenoviruses was verified by qRT-PCR with *GAPDH* as the internal reference gene. (Figure 5; *p* < 0.05)

## 4. Discussion

Adenovirus is a viral vector widely used in scientific research such as gene expression research and gene therapy. PLIN1 acts as a scaffold on the surface of LD and has a structural and regulatory role in the formation and function of LD [34]. Lipid droplets are dynamic organelles in the adipocytes. Under basic conditions, PLIN1 can prevent the breakdown of basic fat and increase lipid synthesis and LD formation [35]. Knocking down the *PLIN1* gene increases basal lipolysis, reduces the size of LD in adipocytes, and leads to resistance to diet-induced obesity in mice [36].

In this study, triglyceride assay and oil red O staining showed increased expression of *PLIN1*, but *PLIN1* silencing inhibited TAG synthesis and LD formation, consistent with previous studies in mice. The cell cycle can be divided into G1 (DNA synthesis preparation), S (DNA synthesis), S2 (division preparation), M phase (division), and G0 (quiescent) [37]. In the flow cytometry test, the cell DNA is stained with the fluorescent dye PI, and the DNA content in the cells was analyzed according to the fluorescence intensity, and then the percentage of the number of cells in G1/G0, S, and G2/M phases is determined [38]. Common methods for studying the cell cycle include BrdU, EdU, and CCK8 staining. DNA replication activity is detected based on the specific reaction between EdU and the fluorescent dye, and the proliferation of cells can be accurately reflected by detecting the EdU label.

In adipocytes, lipid droplets mainly store triglycerides and cholesterol esters, which hydrolyze when fatty acid levels are exhausted. HSL is an important enzyme involved in the hydrolysis of triglycerides (lipolysis) [39]. Studies have shown that HSL is highly expressed in adipocytes and has hydrolytic activity against triglycerides [40]. ATGL hydrolyzes triglycerides (TG) to diacylglycerol (DAG), while HSL hydrolyzes diacylglycerol DAG to monoacylglycerol (MAG). *PLIN1* and PKA (protein kinase A) can directly or indirectly regulate the activity of HSL and ATGL [41]. PKA-mediated phosphorylation of HSL induces the docking of HSL with lipid droplet monolayers and then tightly binds with *PLIN1* [42]. In this study, overexpression of *PLIN1* significantly downregulate ATGL expression in terms of mRNA and protein, indicating that *PLIN1* has an inhibitory effect on lipolysis at the level of gene transcription and protein translation.

The lipolysis processing of triglycerides in adipocytes is mainly governed by three factors: Lipase, lipid droplet coating proteins, and the lipolysis pathway. As the decisive factor for triglyceride hydrolysis, lipases mainly include adipocyte triglyceride lipase (ATGL), hormone-sensitive lipase (HSL) and monoacylglyceride lipase. The main marker genes in each stage of the fat differentiation process were selected to detect changes in their mRNA and protein expression levels. After overexpression of *PLIN1*, the mRNA expression levels of *ACC*, *FASN*, *PPARγ*, *DGAT2*, *FABP4*, and *LPL* were significantly increased, and the amount of FAS and PPARγ proteins also elevated. On the contrary, after interfering with the *PLIN1* gene, *ACC*, *FASN*, *PPARγ*, *DGAT2*, *FABP4*, and *LPL* mRNA expression decreased, and ACC and FAS protein levels also decreased. Overexpression of *PLIN1* suppresses the expression of *ATGL* mRNA, in contrast, it interferes with the *PLIN1* gene, the mRNA and protein expression of ATGL rise.

The transcriptome is the sum of all RNA transcribed in the cell, including mRNA, non-coding RNA and small RNAs. Transcriptomics primarily involves studies of gene function at the RNA level, and plays an essential part in uncovering the molecular mechanisms of cells and tissues, as well as growth and development, and disease research [43]. Transcriptome sequencing technology is based on second-generation sequencing technology, which mainly analyzes gene expression levels, differential genes, new genes, and gene function annotations. It is an important means of gene function and gene expression. The development of second-generation sequencing technology has offered new approaches and approaches for functional genomics research [44]. RNA-seq has many advantages. It can detect known genes and predict new transcripts without designing probes and needing to know much information, with higher sensitivity and greater dynamic range [45].

The adipogenic differentiation of adipocytes is mainly divided into four stages: the first stage involves MSC differentiation into adipocytes, and the marker gene of this stage is *LPL*; in the second stage, adipocytes undergo growth inhibition with clonal proliferation, thereby forming precursor adipocytes. At this time the cells just begin to appear with fat droplets, and the marker genes are *ADD1* and *C/EBPβ*; the third stage with respect to precursor adipocytes after growth, proliferation, and gene expression includes formation of immature adipocytes. At this time, the cells have a large number of small lipid droplets. The third stage marker genes are *C / EBPα* and *PPARγ*.In relation to the deposition of fat in the cells, small lipid droplets converge into large lipid droplets to fill the fat cells, forming mature fat cells [46]. The fourth stage marker genes are *GPD* (glycerol triphosphate dehydrogenase), *FAS* (fatty acid synthase), *ACC* (acetyl coenzyme A decarboxylase), *FABP4* (fatty fatty acid binding protein), *PCK1* (Phosphoenolpyruvate Carboxykinase 1). With the transcriptome sequencing results, after overexpression of *PLIN1*, the expression levels of key genes *FABP4*, *FAS*, *DGAT2*, and *ACC* all increased during adipocyte differentiation. FABP4 is a fat-derived factor that is significantly upregulated during the differentiation of precursor adipocytes into adipocytes [47]. During the differentiation of precursor adipocytes, Hoxa5 inhibits the PKA/HSL signaling pathwayby regulating FABP4 and therefore promoting adipocyte differentiation and diminishing lipolysis [48].

The PAT family proteins PLIN1 and PLIN2 have the same and opposite regulatory effects on lipolysis in Drosophila, indicating the important role and complex functions of PAT family proteins during the lipolysis process [49]. In bovine adipocytes, the expression level of *PLIN2* decreased and the expression level of *PLIN3* gene increased after overexpression of *PLIN1* gene according to RNA-Seq results. These findings indicate that PAT in bovine adipocytes is consistent with the studies in Drosophila family proteins, such that they not only have the same role in regulating lipid metabolism, but also exert complex opposite regulatory effects among PAT family proteins.

Exploring the genetic variation of candidate genes is an important method to promote Qinchuan beef cattle sub-marker breeding and crucial for economic correlation analysis. Our study shows that *PLIN1* gene has a regulatory role in adipocyte differentiation and lipid metabolism, and a large number of new transcripts have also been detected in different processed adipocyte samples, which provides resources for further improvement of bovine genome annotation and molecular breeding research of beef cattle.

## 5. Conclusions

In bovine adipocytes, overexpression of *PLIN1* gene can promote the expression of fat metabolism-related genes at the mRNA and protein levels. Furthermore, overwxpression of *PLIN1* shows that the number of lipid droplets in fat cells, their volume, and the content of triglyceride increased. The *PLIN1* gene can promote the accumulation of triglycerides in bovine preadipocytes and has an important regulatory role in fat metabolism. RNA-seq was used to analyze differentially expressed genes in bovine preadipocytes after overexpression of adenoviruses Ad-PLIN1 and Ad-NC (empty virus). A total of 1923 differentially expressed genes were detected. The DEGs were enriched in members of the AMPK, Wnt, and PPAR signaling pathways related to fat proliferation and differentiation. This indicates that the *PLIN1* gene has an important regulatory impact on fat proliferation and metabolism. Moreover, the sequencing results also screened new DEGs related to fat metabolism pathways, providing theoretical support for the molecular breeding of Qinchuan beef cattle.

## Figures and Tables

**Table 1:** Exact shRNA and sh-NC sequences for the *PLIN1* mRNA.

Name of shRNAs	Sense Strand (5′–3′)	Loop	Anti-Sense Strand (5′–3′)	RNA Poly III Terminator
shRNA-264	GCCTCTGTATGCAATGCCTAC	TCAAGAG	GTAGGCATTGCATACAGAGGC	TTTTTT
shRNA-786	GCCAACACTCTCTCTAGACAC	TCAAGAG	GTGTCTAGAGAGAGTGTTGGC	TTTTTT
shRNA-1337	GATGGAACCCGAGAGCGAATT	TCAAGAG	GAATTCGCTCTCGGGTTCCATC	TTTTTT
shRNA-NC	GTTCCACGACCAAATCAGCTC	TCAAGAG	GAGCTGATTTGGTCGTGGAAC	TTTTTT

**Table 2:** Gene expression analysis, and differentially expressed genes Identification.

Gene Name	Gene Full Name	log2FoldChange (Ad/NC)	*p*-Value
*ACSF2*	acyl-CoA synthetase family member 2	1.579670955	1.69E−221
*RBP4*	retinol binding protein 4	0.775424841	6.02E−46
*THBS3*	thrombospondin 3	0.828570053	3.32E−59
*LUM*	lumican	0.720545336	1.99E−101
*GLIPR2*	GLI pathogenesis related 2	0.845982741	1.59E−96
*CSF1*	colony stimulating factor 1	1.252623086	1.33E−241
*SYTL2*	synaptotagmin like 2	0.798285899	2.66E−132
*NR4A1*	nuclear receptor subfamily 4 group A member 1	0.726639325	2.10E−33
*PLTP*	phospholipid transfer protein	2.733646178	6.32E−256
*PALLD*	palladin, cytoskeletal associated protein	0.852884845	2.09E−132
*TMEM79*	transmembrane protein 79	−0.864004155	6.77E−16
*CYR61*	cysteine rich angiogenic inducer 61	0.961861819	1.90E−154
*ZFP36L1*	ZFP36 ring finger protein like 1	−0.455224558	3.95E−29
*SPOCK2*	SPARC/osteonectin, cwcv and kazal like domains proteoglycan 2	−1.811236856	9.02E−173
